# Chronic Microcystin-LR Exposure Induces Abnormal Lipid Metabolism via Endoplasmic Reticulum Stress in Male Zebrafish

**DOI:** 10.3390/toxins12020107

**Published:** 2020-02-07

**Authors:** Dandan Zhang, Wang Lin, Yinjie Liu, Honghui Guo, Lingkai Wang, Liping Yang, Li Li, Dapeng Li, Rong Tang

**Affiliations:** 1 College of Fisheries, Huazhong Agricultural University, Wuhan 430070, China; dan19950824@webmail.hzau.edu.cn (D.Z.); linwang@webmail.hzau.edu.cn (W.L.); lyinjie650@gmail.com (Y.L.); honghuiguo@webmail.hzau.edu.cn (H.G.); wanglingkai@webmail.hzau.edu.cn (L.W.); yangliping@webmail.hzau.edu.cn (L.Y.); ldp@mail.hzau.edu.cn (D.L.); tangrong@mail.hzau.edu.cn (R.T.); 2 Hubei Provincial Engineering Laboratory for Pond Aquaculture, College of Fisheries, Huazhong Agricultural University, Wuhan 430070, China; 3 National Demonstration Center for Experimental Aquaculture Education, Huazhong Agricultural University, Wuhan 430070, China

**Keywords:** microcystin-LR, zebrafish, endoplasmic reticulum stress, lipid metabolism

## Abstract

In order to explore effects of low levels of continuous microcystin-LR (MC-LR) (a cyanotoxin) exposure on hepatic lipid metabolism on the basis of the endoplasmic reticulum stress (ERS) pathway, we exposed adult male zebrafish to MC-LR (0, 1, 5, and 25 μg/L) for 60 days, and hepatic histopathology as well as lipid metabolic parameters were determined with mRNA levels of ERS signal molecules and downstream factors, along with genes associated with lipid metabolism in zebrafish liver. The results revealed that prolonged exposure to MC-LR remarkably altered the levels of hepatic total cholesterol and triglyceride and led to hepatic steatosis, which was also confirmed by hepatic cytoplasmic vacuolization in Hematoxylin/eosin (H&E) stain and lipid droplet accumulation in Oil Red O stain. The severity of hepatic damage and lipidation was increased in a dose-related manner. MC-LR exposure significantly upregulated transcriptional levels of ERS markers including *hspa5*, *mapk8*, and *chop*, indicating the occurrence of ERS in the liver of zebrafish. Concurrently, MC-LR significantly improved mRNA expression of unfolded protein response (UPR) pathway-related genes including *atf6*, *eif2ak3*, *ern1*, and *xbp1s*, suggesting that all of the three UPR branches were activated by MC-LR. MC-LR also induced significant upregulation of downstream lipid metabolism-related factors and genes including *srebf1*, *srebf2*, fatty acid synthase (*fasn*), acetyl-CoA carboxylase (*acaca*), stearoyl-CoA desaturase (*scd*), HMG CoA reductase (*hmgcra*), and HMG CoA synthase (*hmgcs1*), and downregulation of genes associated with lipolysis such as triglyceride hydrolase gene (*atgl*), hormone-sensitive enzyme gene (*hsla*), and carnitine palmitoyltransferase gene (*cpt1aa*). Our present results indicated that the cause of hepatic lipid accumulation by MC-LR was mainly by upregulating lipogenic and cholesterol genes but downregulating the expression of lipolytic genes through the induction of *srebf1* and *srebf2*, which were involved in the activation of ERS signal pathways.

## 1. Introduction

Due to combined effects of various factors such as water eutrophication and global warming, more and more freshwater lakes around the world are experiencing severe cyanobacterial blooms, such as Lake of the Woods in Canada [1]; Lake Erie in the USA [2]; Lake Zegerplas in the Netherlands [3]; Peel–Harvey estuaries in Western Australia [4]; and Lakes Poyang, Chaohu, and Dianchi, as well as Erhai in China [5,6,7]. The frequent occurrence of cyanobacterial blooms not only deteriorates water quality but also produces highly toxic microcystins (MCs). MCs can also be deposited in sediments; the production of these toxins may also occur directly in sediments, and some deposited MCs can be released back go water column [8]. Currently, MC pollution has become a global public health concern as MCs have been reported to be involved in numerous cases of lethal poisonings in wild animals, livestock, and even humans [9,10,11]. More than 200 MC analogues have been confirmed to date [12]. Among MCs such as microcystin-LR (MC-LR), microcystin-RR (MC-RR), and microcystin-YR (MC-YR), MC-LR is known as the most toxic and most widely distributed variant [13]. In 2010, the International Agency for Research on Cancer (IARC) classified MC-LR as “probably carcinogenic to humans” (group 2B carcinogens) [13]. It is important to realize that although the World Health Organization’s recommended level of MC-LR in the environment is 1 μg/L, actual environmental concentration of MC-LR often exceeds the standard [14,15].

Accumulating evidence has shown that the main target organ of MCs is the liver, and exposure to high levels of MCs can cause acute liver injury, which is characterized by severe intrahepatic hemorrhage, disruption of liver cords, and hepatocyte necrosis and apoptosis [16,17,18]. The putative mechanisms of action of MC hepatotoxicity include the specific inhibition of protein serine/threonine phosphatases types 1 and 2A, oxidative stress, and DNA damage [16,18,19,20]. In fact, waterborne MC-LR can last in aqueous solution up to several months and even a full year with the outbreak of blooms [15], posing a high hazard to ecological and public health. The epidemiological investigations also demonstrated that many chronic health problems such as hepatitis, liver lipid peroxidation, and liver cancer could be induced in animals or people who receive low levels of MCs for a long term via immersion or drinking water [10,21,22]. Recently, a few omics studies suggested that low levels of MC-LR could cause hepatic lipid metabolism abnormalities in fish and mammals [23,24]; however, the potential mechanism behind this effect remains unclear.

In cells, the endoplasmic reticulum (ER) is found to be a major site of action of MCs [25]. Interestingly, ER is known to be a critical organelle that regulates lipid anabolism and catabolism, protein folding, as well as calcium homeostasis [26,27]. Various condition changes including hypoxia, viral infection, and other toxic insults can cause an accumulation of non- and misfolded proteins in the ER lumen, which in turn induces ER stress (ERS) and subsequent unfolded protein response (UPR) [28]. In order to facilitate protein folding and maintain homeostasis of the ER during stress, the UPR can activate a unique signaling network via three pathways, the protein kinase RNA-activated-like ER kinase (PERK)-eukaryotic translation initiation factor 2 alpha (eIF2α) pathway, the activating transcription factor 6 (ATF6) pathway, and the inositol-requiring enzyme 1 (IRE1α)-X-box binding protein 1 (XBP1) pathway [29]. Some studies have shown that MC-LR can induce tissue damage or cell apoptosis through the ERS pathway in mammals [30,31,32,33]. Nevertheless, the potential role of ERS response in MC-LR-induced lipid metabolism abnormalities has not yet been addressed. It is well accepted that ERS can trigger activation of sterol regulatory element binding proteins (SREBPs) [34,35], which is in charge of the regulation of related genes that are involved in lipogenesis/cholesterogenesis and lipolysis. In mammals, increasing studies have demonstrated that ERS plays a crucial role in hepatic steatosis by mediating the transcriptional regulation of fatty acid biosynthesis via SREBP-1c, a member of the SREBPs family [36,37,38]. As known to all, lipids serve as a main energy source and participate in a variety of physiological processes in teleost fish, and thus abnormalities in lipid metabolism contribute significantly to the morbidity and mortality when fish are exposed to waterborne bacteria, virus, and pollutants [39]. Therefore, it is very imperative to explore how ERS is involved in the modulation of the lipid metabolism disorder caused by MC-LR in fish liver.

Given the above reasons, we investigated the effects of low levels of continuous MC-LR (0, 1, 5, and 25 μg/L) exposure on liver lipid metabolism in adult zebrafish, as well as the potential mechanism of the ERS in MC-LR-induced hepatic lipid metabolism disturbance. Thus, in our study, hepatic histopathology and lipid metabolic parameters as well as transcriptional levels of ERS-related molecules were determined in adult zebrafish, along with mRNA expression of downstream lipid metabolism-related transcriptional factors and genes. The results provide a novel insight to the mechanisms associated in MC-LR-induced hepatotoxicity in fish.

## 2. Results

### 2.1. H&E and Oil Red-O Stain

**Figure 1:**
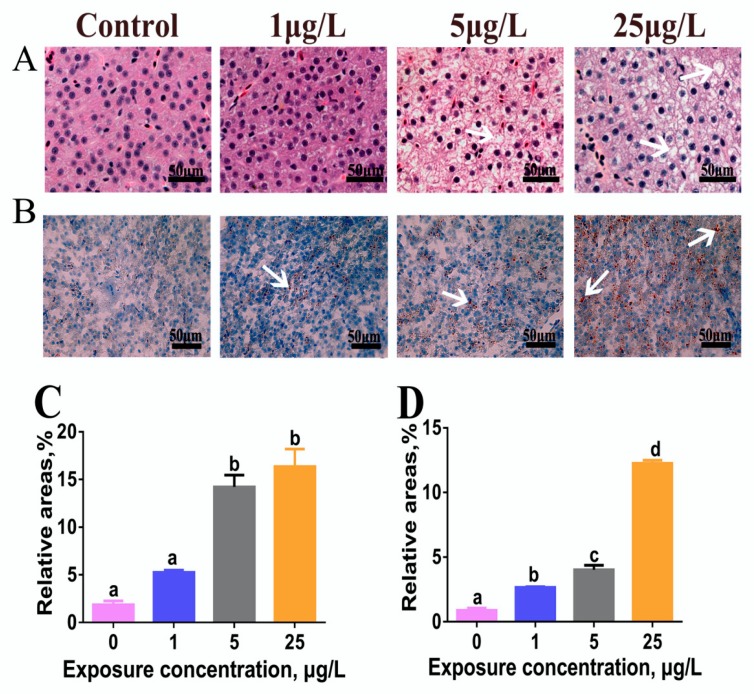
Microcystin-LR (MC-LR) influences on hepatic lipid accumulation in adult male zebrafish exposed to low levels of MC-LR (0, 1, 5, and 25 µg/L) for 60 days. (**A**) Liver histology (hematoxylin/eosin (H&E) stain) and (**B)** histochemistry (Oil Red O stain). Arrows indicate hepatic steatosis. Lipid droplets are red-colored and nuclei are blue-colored with Oil Red O stain. (**C**,**D**) The relative areas for liver vacuoles in H&E stain and lipid droplets in Oil Red O stain were analyzed by Image-Pro plus 6.0. Values are reported as mean ± standard error (SE) (*n* = 5) and normalized to percentage of field area. Significant differences at *p* < 0.05 are indicated by different letters above bars.

As shown in Figure 1A, the amount of hepatic cytoplasmic vacuolation in MC-LR-treated zebrafish was increased significantly in a dose-related manner (Figure 1A). MC-LR exposure also elevated the amount of lipid droplets in the liver of zebrafish (Figure 1B). Furthermore, these findings were confirmed by the areas quantified for hepatic vacuoles in hematoxylin/eosin (H&E) stain and lipid drops in Oil Red O stain (Figure 1C,D).

### 2.2. Total Cholesterol and Triglyceride Levels in Liver Tissue

**Figure 2:**
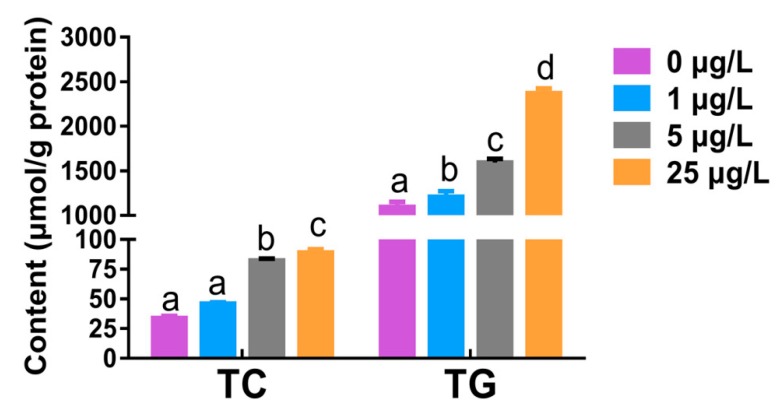
The contents of total cholesterol (TC) and triglyceride (TG) in the liver of adult zebrafish exposed to 0, 1, 5, and 25 μg/L MC-LR for 60 days. Values are reported as mean ± SE (*n* = 5). Significant differences at *p* < 0.05 are indicated by different letters above bars.

Compared with the control group, hepatic total cholesterol (TC) levels were significantly elevated 2.4-fold and 2.6-fold in the fish treated with 5 and 25 μg/L MC-LR (*p* < 0.05), respectively. Similarly, total triglyceride (TG) levels were remarkably increased in a level-dependent manner (*p* < 0.05), and the maximal increase was up to 2.5-fold in the 25 μg/L MC-LR group relative to the control (Figure 2).

### 2.3. Marker Gene Transcription of ERS-UPR Pathways

**Figure 3:**
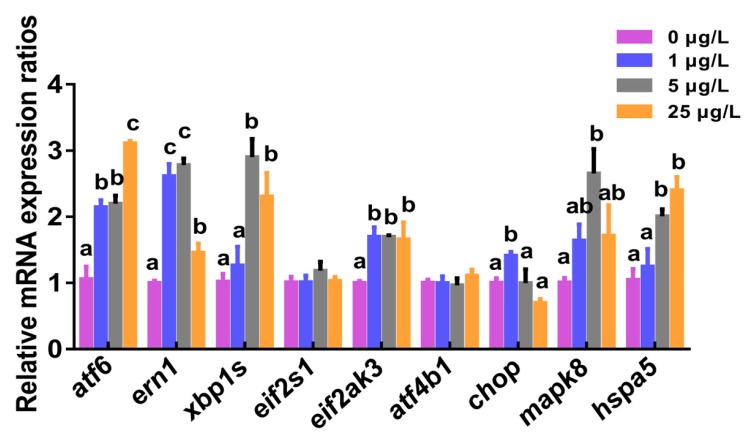
The relative mRNA expression of endoplasmic reticulum stress (ERS) signaling molecules in the liver of adult zebrafish exposed to 0, 1, 5, and 25 μg/L MC-LR for 60 days. Values are reported as mean ± SE (*n* = 5). Significant differences at *p* < 0.05 are indicated as different letters above bars.

Figure 3 showed changes in transcriptional levels of marker genes of the ERS and three UPR signaling pathways in zebrafish liver induced by MC-LR. Chronic MC-LR exposure caused significant increases in mRNA levels of hepatic ERS signaling molecules (*chop*, *mapk8*, and *hspa5*) (*p* < 0.05). Compared to the controls, transcriptional levels of marker genes *atf6*, *ern1*, *xbp1s*, and *eif2ak3* in the three UPR signaling pathways showed concentration-course increases (*p* < 0.05) and the maximal increases of *atf6*, *ern1*, *xbp1s*, and *eif2ak3* mRNAs were up to 3.1-fold, 2.8-fold, 2.9-fold, and 1.7-fold, respectively. There were no significant differences for mRNA expression levels of hepatic *eif2s1* and *atf4b1*.

### 2.4. Transcription of Lipid Metabolism-related Genes

**Figure 4:**
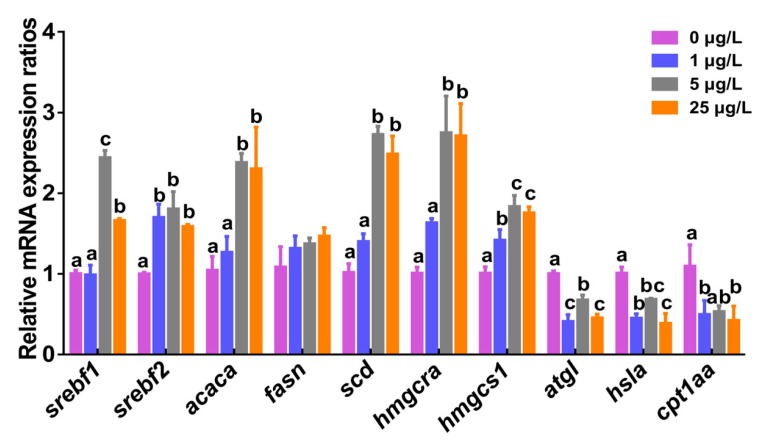
The relative mRNA expression of lipid metabolism-related genes in the liver of zebrafish exposed to 0, 1, 5, and 25 μg/L MC-LR for 60 days. Values are reported as mean ± SE (*n* = 5). Significant differences at *p* < 0.05 are indicated by different letters above bars.

**Figure 5:**
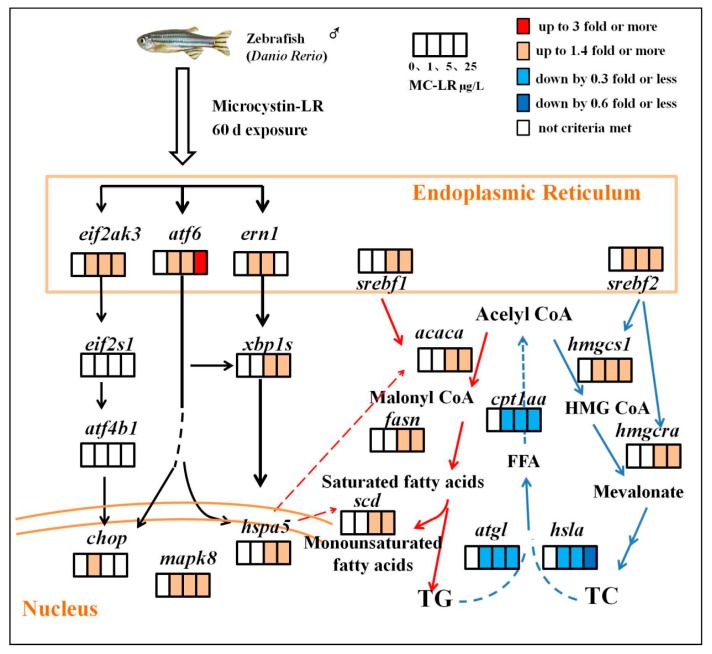
Graphical summary of hepatic lipid disturbance induced by MC-LR via the ERS pathway in zebrafish.

To study if ERS induced by MC-LR might influence hepatic lipid metabolism including fatty acid metabolism and cholesterol metabolism, transcriptional levels of lipid metabolism-related factors and genes were examined in this study (Figure 4 and Figure 5). More detailed descriptions about transcriptional levels of genes after exposure to MC-LR are provided in the Supplementary Materials section (Table S1). The mRNA expression of *srebf1* in zebrafish liver were remarkably upregulated 2.4- and 1.7-fold in 5 and 25 μg/L MC-LR exposure groups relative to the control group (*p* < 0.05), respectively. Similarly, hepatic *srebf2* mRNA was significantly increased 1.7-, 1.8-, and 1.6-fold in three MC-LR exposure groups. The mRNA levels of both fatty acid synthesis-related genes (acetyl-CoA carboxylase (*acaca*) and stearoyl-CoA desaturase (*scd*)) genes and cholesterol synthesis-related genes (HMG CoA reductase (*hmgcra*) and HMG CoA synthase (*hmgcs1*)) were significantly up-regulated in 5 and 25 μg/L MC-LR exposure groups (*p* < 0.05). No significant difference was detected for gene expression of hepatic fatty acid synthase (*fasn*). Compared with the control, transcriptional levels of triglyceride hydrolase gene (*atgl*), hormone-sensitive enzyme gene (*hsla*), and carnitine palmitoyltransferase gene (*cpt1aa*) related to lipolysis were markedly down-regulated in the three MC-LR exposure groups (*p* < 0.05).

## 3. Discussion

In the natural environment, aquatic organisms are continuously exposed to MCs for a long period of time, and some even throughout their life cycle. However, previous studies have generally focused on exploring the hepatotoxic effects of MCs under acute exposure [17,18,40,41]. This study, through chronic MC-LR exposure, brought new mechanistic information on ERS involved in the modulation of hepatic lipid metabolism disorder in fish.

In the present study, histological and histochemical findings showed that chronic MC-LR exposure caused a level-dependent lipid deposition in the liver of zebrafish, which was characterized with hepatic cytoplasmic vacuolization in H&E stain and lipid droplet accumulation in Oil Red O stain. Consistent with this, marked increases in the levels of hepatic TC and TG further confirmed this result. In fact, liver steatosis is a vital indicator of abnormal lipid metabolism in liver disease [42]. Similarly, Li et al. [43] observed an increase of hepatic lipid droplets in the number and size in common carp fed with *Microcystis* bloom scum (50 μg MC-LR/kg body weight) under laboratory conditions for 28 days. Liver lipid accumulation was also found in silver carp that were cultured in a large pen located in Meiliang Bay of Taihu Lake, China, for a year [44].

Hspa5, a zebrafish homolog of ER molecular chaperone GRP78, can bind transmembrane sensors such as IRE1, PERK, and ATF6, and keep them inactive during unstressed conditions [45,46]. When misfolded or unfolded proteins accumulate in the ER, Hspa5 leaves these sensors to carry out its chaperone function to activate the UPR signal. CHOP is an ERS-induced transcription factor, which is associated with cell apoptosis [47]. MAPK8 is a zebrafish homolog of JNK and often acts as a crucial mediator of apoptosis. In the present study, chronic MC-LR exposure significantly upregulated transcriptional levels of ERS markers including *hspa5*, *mapk8*, and *chop*, indicating the occurrence of ERS in the liver of zebrafish. In in vitro human hepatoma cells (Huh7), MC-LR has been documented as causing ERS and the activation of all of three UPR signaling pathways [32]. Also, ERS induction was reported in the liver, kidney, and brain of in vivo mammals exposed to MC-LR [30,33]. In our study, a significant transcriptional introduction of hepatic *atf6*, *ern1*, and *eif2ak3* was concurrently detected in three MC-LR-treated groups, which further revealed that all of three UPR branches were activated by low levels of continuous MC-LR exposure. By contrast, the increases in levels of *atf6* and *ern1* mRNAs were higher than that of *eif2ak3* in our study. Meanwhile, our results showed that MC-LR resulted in a marked induction of *xbp1s*, which was proven to be a key regulator in enhancing ER folding capacity and in dealing with ERS [36]. In general, *xbp1s* activation is able to initiate a transcriptional program either alone in the IRE1α-XBP1 pathway or in combination with *atf6α* in the ATF6 pathway, which will subsequently generate ERS molecular chaperones (such as *hspa5*) that help protein folding to relieve ERS. Thus, in this study, the significant upregulation of *xbp1s* mRNA might imply that the two UPR pathways, the ATF6 pathway and the IRE1α-XBP1 pathway, play a major role in ERS induced by MC-LR exposure.

There is a close relationship between ERS and hepatic lipid metabolism, as ERS participates in liver lipogenesis by controlling mRNA expression of genes in the adipogenic pathway [28,48,49]. Using mRNA-Seq and qPCR analyses, Howarth et al. [50] reported that *atf6* could interact with *fasn* to cause alcoholic fatty liver disease (FLD), suggesting that triglyceride biogenesis induced by UPR is a mechanism of FLD. Of course, the mechanism by which ERS stimulates lipogenesis is multifactorial. The sterol regulatory element binding protein (SREBP) family is the most important transcription factor present in the ER, which is involved in the regulation of target genes associated with the synthesis of lipid (such as SREBP1c) and cholesterol metabolism (such as SREBP1a and SREBP2) [51]. Passeri et al. [52] documented that acute alcohol exposure caused hepatic SREBP activation in zebrafish, thereby inducing gene expression required for de novo lipid and cholesterol synthesis and leading to liver steatosis. SREBP1c can activate enzymes related to fatty acid biosynthesis and lipogenesis, and targeting enzyme genes include fatty acid synthase (*fasn*), acetyl-CoA carboxylase (*acaca*), and stearoyl-CoA desaturase (*scd*) [32,49,51,52,53]. In the present study, we found that the mRNA expression of *srebf1*, a zebrafish homolog of SREBP1c, was elevated significantly in 5 and 25 μg/L MC-LR exposure groups. Furthermore, its regulated downstream factors *acaca*, *fasn*, and *scd* were up-regulated accordingly, suggesting increased lipid synthesis in zebrafish liver. The present study also indicated that mRNA levels of these lipogenic genes (*acaca*, *fasn*, and *scd*) paralleled alterations of hepatic lipid contents. Similar results were reported in previous studies on copper-induced hepatic steatosis [54,55,56]. Lee et al. [36] reported that elevated gene expression of XBP1 in mice after being fed carbohydrates were accompanied with the induction of decisive genes related to fatty acid synthesis. Cao et al. [57] also documented that a high-fat diet could cause abnormal lipid secretion by activating the ERS-associated IRE1/XBP1 pathway. Apparently, the marked upregulation of *xbp1* in our study might enhance hepatic lipogenesis and lead to subsequent liver steatosis. In addition, the activation of SREBP2 can induce genes required for cholesterol production including HMG CoA reductase (*hmgcra*) and HMG CoA synthase (*hmgcs1*) [58]. In our present study, mRNA levels of hepatic *srebf2* and downstream factors *hmgcra* and *hmgcs1* were significantly up-regulated in MC-LR-treated groups, suggesting that MC-LR might stimulate the production of cholesterol by the induction of *srebf2*. Experiments in cultured HK-2 cells have shown that CsA-induced ERS results in increased SREBP-2 activation, leading to lipid accumulation and apoptosis [59]. Also, Rong et al. [60] documented that SREBP-2 mediates the expression of cholesterol biosynthetic genes and controlled steady-state tissue cholesterol levels by regulating the uptake and synthesis of cholesterol in mice. In this regard, our results suggested chronic MC-LR exposure enhanced the production of fatty acids and cholesterol through the induction of *srebf1* and *srebf2.* On the other hand, transcriptional levels of genes associated with lipolysis such as the triglyceride hydrolase gene (*atgl*), the hormone sensitive enzyme gene (*hsla*), and the carnitine palmitoyltransferase gene (*cpt1aa*) were significantly down-regulated in this study, which implied a decrease in lipolysis. The result was further supported by the changes in the levels of TC. One of the most serious causes of abnormal lipid metabolism is the imbalance between hepatic lipogenesis and lipolysis [61]. Therefore, our experimental results indicated that the cause of hepatic lipid accumulation by MC-LR was mainly by upregulating lipogenic and cholesterol genes but downregulating the expression of lipolytic genes through the induction of *srebf1* and *srebf2*, which were involved with the activation of ERS signal pathways. Our research still has some limitations, for example, MC-LR may be adsorbed in the natural environment and thus the exposure of fish may be limited; the presence of other compounds in the natural environment can at least partially reduce the toxicity of MC-LR and the like.

## 4. Conclusions

Our present results provide evidence that chronic MC-LR exposure induces ERS and SREBP activation, which subsequently drives the expression of hepatic lipid and cholesterol metabolism-related genes, leading to increased hepatic lipogenesis and steatosis. These findings provide a mechanistic insight for the hepatotoxicity of MC-LR, and can be extrapolated to the potential impacts of MCs on human health.

## 5. Materials and Methods

### 5.1. Reagents

MC-LR (purity ≥95% confirmed by HPLC) was purchased from Express (Express Technology Co. Ltd., Taiwan), and then dissolved into MilliQ water to prepare a stock solution with a centration of 0.5 mg/mL. RNAiso reagent, PrimeScript RT reagent kit (Perfect Real Time, Roche, Basel, Switzerland), and iQ^TM^ SYBR^®^ Green Supermix were bought from TaKaRa (Dalian, China). It was ensured that all the reagents utilized in this experiment were of analytical grade. 

### 5.2. Adult Fish Maintenance and MC-LR Treatment

Wild-type adult male zebrafish (AB strain) were purchased from the Institute of Hydrobiology, Chinese Academy of Sciences, and cultured at 28 °C in flow-through tanks with carbon filtered tap water under 14:10 h (light/dark). The *Artemia nauplii* were fed to the fish twice a day to ensure the normal growth and nutrition of fish. After a 14 day acclimation period, fish were exposed to MC-LR at levels of 0, 1, 5, or 25 μg/L for 60 days. Each concentration was performed in three replicate tanks, and each tank was assigned randomly for 35 fish. Exposure concentrations were set on the basis of our previous experiments, and on environmental relevance [15,62,63]. During the exposure period, one-third of exposure solution was replaced every 3 days with the corresponding MC-LR or MC-LR-free fresh water, and the actual MC-LR levels were measured by the commercial ELISA kit for MC-LR (Beacon Analytical Systems, Inc., Saco, ME, USA).

After 60 days of exposure, fish were firstly euthanized with 0.02% tricaine methanesulfonate (MS-222), and then liver tissues were removed immediately. Partial liver samples were frozen with liquid nitrogen and stored in a −80 °C refrigerator until later analysis. The other liver samples were fixed for histological evaluation. All procedures in this study were approved according to the guidelines of the Institutional Animal Care and Use Committee (IACUC) of Huazhong Agricultural University (permission number: HZAUFI-2018-015, date of approval: 6 April 2018), Wuhan, China.

### 5.3. Histological and Histochemical Examination

For histological assessment, samples were fixed with 10% neutral formalin buffer, routinely processed, sectioned at 5 μm after paraffin embedding, and then stained with hematoxylin/eosin (H&E). As for histochemical observation, liver samples were directly sliced in a frozen microtome, and the obtained frozen sections were stained with Oil Red O, mounted tablets, and then prepared for optical microscopy. Histological and histochemical evaluations were performed on a Nikon H600L microscope and the image analysis system (Tokyo, Japan). The relative areas of liver vacuoles in H&E stain and lipid droplets in Oil Red O stain were counted by randomly examining 10 microscope fields for each sample, and 5 individual samples for each treatment were used for calculation.

### 5.4. Biochemical Indicator Detection

Liver samples were homogenized in absolute ethanol (1:10 w/v), and the homogenate was directly used as the assay source. The levels of total cholesterol (TC) and triglyceride (TG) were detected by the COD-PAP and GPO-PAP (cholesterol oxidase/glycerol-3-phosphate oxidase coupled to phenol and 4-aminophenazone) methods, using the commercial kits from Nanjing Jiancheng Bioengineering Institute, Nanjing, China. With reference to Bradford [64], protein contents of liver homogenates were measured using bovine serum albumin (BSA) as standard. The contents of TC and TG were expressed as micromole per gram protein. All these assays were run in triplicate.

### 5.5. qPCR Analysis

Liver samples were homogenized in RNAiso Plus (TaKaRa, Dalian, China) for extracting total RNA as described by the manufacturer. RNA concentration and purity were assessed by determining the 260 nm absorbance and 260/280 ratios. For each sample, 1 μg of RNA was reverse transcribed to cDNA using Takara PrimeScript RT reagent kit with gDNA Eraser according to the manufacturer’s protocol. qPCR was actualized using Takara SYBR Green Kits on the iQ5 Multicolor Real-Time PCR Detection System (Bio-Rad Laboratories, Hercules, CA, USA). Gene-specific primers were designed using Primer-BLAST (Table S1). The PCR protocol in this study was set as follows: pre-denaturation at 95 °C for 30 s, 40 cycles of denaturation at 95 °C for 10 s, annealing at 58 °C for 20 s, and elongation 72 °C for 20 s. On the basis of the studies by Lin et al. [62] and Hou et al. [63], we selected glyceraldehyde-3-phosphate dehydrogenase (*gapdh*) as the internal control in this study to normalize all data. The relative expression of target genes was calculated using the 2^−ΔΔCt^ method [65].

### 5.6. Statistical Analysis

SPSS 22.0 for Windows was applied to statistical analysis. Values are expressed as mean ± standard error (SE). The significant differences were analyzed by one-way ANOVA followed by Duncan’s test. Normality and variance uniformity were verified. Statistical significance was defined at *p* < 0.05.

## Supplementary Materials

The following are available online at https://www.mdpi.com/2072-6651/12/2/107/s1: Table S1: Transcriptional levels of genes after exposure to MC-LR. Table S2: Sequences of primers for tested genes.

## References

[1] Chen H., Burke J.M., Dinsmore W.P., Prepas E.E., Fedorak P.M. (2007). First assessment of cyanobacterial blooms and microcystin-LR in the Canadian portion of Lake of the Woods. Lake Reserv. Manag..

[2] Rinta-Kanto J.M., Konopko E.A., DeBruyn J.M., Bourbonniere R.A., Boyer G.L., Wilhelm S.W. (2009). Lake Erie Microcystis: Relationship between microcystin production, dynamics of genotypes and environmental parameters in a large lake. Harmful Algae.

[3] Via-Ordorika L., Fastner J., Kurmayer R., Hisbergues M., Dittmann E., Komarek J., Erhard M., Chorus I. (2004). Distribution of microcystin-producing and non-microcystin-producing Microcystis sp. in European freshwater bodies: Detection of microcystins and microcystin genes in individual colonies. Syst. Appl. Microbiol..

[4] Kemp A., John J. (2006). Microcystins associated with Microcystis dominated blooms in the southwest wetlands, Western Australia. Environ. Toxicol. Int. J..

[5] Yu L., Kong F., Zhang M., Yang Z., Shi X., Du M. (2014). The dynamics of Microcystis genotypes and microcystin production and associations with environmental factors during blooms in Lake Chaohu, China. Toxins.

[6] Wu Y., Li L., Gan N., Zheng L., Ma H., Shan K., Song L. (2014). Seasonal dynamics of water bloom-forming Microcystis morphospecies and the associated extracellular microcystin concentrations in large, shallow, eutrophic Dianchi Lake. J. Environ. Sci..

[7] Zhang D., Liao Q., Zhang L., Wang D., Luo L., Chen Y., Zhong J., Liu J. (2015). Occurrence and spatial distributions of microcystins in Poyang Lake, the largest freshwater lake in China. Ecotoxicology.

[8] Henao E., Rzymski P., Water M.N. (2020). A Review on the Study of Cyanotoxins in Paleolimnological Research: Current Knowledge and Future Needs. Toxins.

[9] Azevedo S.M., Carmichael W.W., Jochimsen E.M., Rinehart K.L., Lau S., Shaw G.R., Eaglesham G.K. (2002). Human intoxication by microcystins during renal dialysis treatment in Caruaru—Brazil. Toxicology.

[10] Zhao Y., Xue Q., Su X., Xie L., Yan Y., Steinman A.D. (2015). Microcystin-LR induced thyroid dysfunction and metabolic disorders in mice. Toxicology.

[11] Rezaitabar S., Sari A.E., Bahramifar N., Ramezanpour Z. (2017). Transfer, tissue distribution and bioaccumulation of microcystin LR in the phytoplanktivorous and carnivorous fish in Anzali wetland, with potential health risks to humans. Sci. Total Environ..

[12] Meriluoto J., Spoof L., Codd G.A. (2016). Appendix 3: Tables of Microcystins and Nodularins. Handb. Cyanobacterial Monit. Cyanotoxin Anal..

[13] Gupta N., Pant S.C., Vijayaraghavan R., Lakshmana Rao P.V. (2003). Comparative toxicity evaluation of cyanobacterial cyclic peptide toxin microcystin variants (LR, RR, YR) in mice. Toxicology.

[14] Lahti K., Rapala J., Färdig M., Niemelä M., Sivonen K. (1997). Persistence of cyanobacterial hepatotoxin, microcystin-LR in particulate material and dissolved in lake water. Water Res..

[15] Wang Q., Niu Y., Xie P., Chen J., Ma Z., Tao M., Qi M., Wu L., Guo L. (2010). Factors affecting temporal and spatial variations of microcystins in Gonghu Bay of Lake Taihu, with potential risk of microcystin contamination to human health. Sci. World J..

[16] Li L., Xie P. (2009). Hepatic histopathological characteristics and antioxidant response of phytoplanktivorous silver carp intraperitoneally injected with extracted microcystins. Biomed. Environ. Sci..

[17] Trinchet I., Djediat C., Huet H., Dao S.P., Edery M. (2011). Pathological modifications following sub-chronic exposure of medaka fish (Oryzias latipes) to microcystin-LR. Reprod. Toxicol..

[18] Hou J., Li L., Xue T., Long M., Su Y., Wu N. (2015). Hepatic positive and negative antioxidant responses in zebrafish after intraperitoneal administration of toxic microcystin-LR. Chemosphere.

[19] MacKintosh C., Beattie K.A., Klumpp S., Cohen P., Codd G.A. (1990). Cyanobacterial microcystin-LR is a potent and specific inhibitor of protein phosphatases 1 and 2A from both mammals and higher plants. FEBS Lett..

[20] Svirčev Z., Baltić V., Gantar M., Juković M., Stojanović D., Baltić M. (2010). Molecular aspects of microcystin-induced hepatotoxicity and hepatocarcinogenesis. J. Environ. Sci. Health Part C.

[21] Chen J., Zhang D., Xie P., Wang Q., Ma Z. (2009). Simultaneous determination of microcystin contaminations in various vertebrates (fish, turtle, duck and water bird) from a large eutrophic Chinese lake, Lake Taihu, with toxic Microcystis blooms. Sci. Total Environ..

[22] Zhang F., Lee J., Liang S., Shum C.K. (2015). Cyanobacteria blooms and non-alcoholic liver disease: Evidence from a county level ecological study in the United States. Environ. Health.

[23] He J., Chen J., Wu L., Li G., Xie P. (2012). Metabolic response to oral microcystin-LR exposure in the rat by NMR-based metabonomic study. J. Proteome Res..

[24] Chen L., Hu Y., He J., Chen J., Giesy J.P., Xie P. (2016). Responses of the proteome and metabolome in livers of zebrafish exposed chronically to environmentally relevant concentrations of microcystin-LR. Environ. Sci. Technol..

[25] Alverca E., Andrade M., Dias E., Bento F.S., Batoréu M.C.C., Jordan P., Pereira P. (2009). Morphological and ultrastructural effects of microcystin-LR from Microcystis aeruginosa extract on a kidney cell line. Toxicon.

[26] Görlach A., Klappa P., Kietzmann D.T. (2006). The endoplasmic reticulum: Folding, calcium homeostasis, signaling, and redox control. Antioxid. Redox Signal..

[27] Babour A., Bicknell A.A., Tourtellotte J., Niwa M. (2010). A surveillance pathway monitors the fitness of the endoplasmic reticulum to control its inheritance. Cell.

[28] Hotamisligil G.S. (2010). Endoplasmic reticulum stress and the inflammatory basis of metabolic disease. Cell.

[29] Wang S., Kaufman R.J. (2012). The impact of the unfolded protein response on human disease. J. Cell Biol..

[30] Qin W., Xu L., Zhang X., Wang Y., Meng X., Miao A., Yang L. (2010). Endoplasmic reticulum stress in murine liver and kidney exposed to microcystin-LR. Toxicon.

[31] Qin W., Zhang X., Yang L., Xu L., Zhang Z., Wu J., Wang Y. (2015). Microcystin-LR altered mRNA and protein expression of endoplasmic reticulum stress signaling molecules related to hepatic lipid metabolism abnormalities in mice. Environ. Toxicol. Pharmacol..

[32] Christen V., Capelle M., Fent K. (2013). Silver nanoparticles induce endoplasmatic reticulum stress response in zebrafish. Toxicol. Appl. Pharmacol..

[33] Cai F., Liu J., Li C., Wang J. (2015). Critical role of endoplasmic reticulum stress in cognitive impairment induced by microcystin-LR. Int. J. Mol. Sci..

[34] Lee J.N., Ye J. (2004). Proteolytic activation of sterol regulatory element-binding protein induced by cellular stress through depletion of Insig-1. J. Biol. Chem..

[35] Colgan S.M., Tang D., Werstuck G.H., Austin R.C. (2007). Endoplasmic reticulum stress causes the activation of sterol regulatory element binding protein-2. Int. J. Biochem. Cell Biol..

[36] Lee A.H., Scapa E.F., Cohen D.E., Glimcher L.H. (2008). Regulation of hepatic lipogenesis by the transcription factor XBP1. Science.

[37] Kammoun H.L., Chabanon H., Hainault I., Luquet S., Magnan C., Koike T., Ferré P., Foufelle F. (2009). GRP78 expression inhibits insulin and ER stress–induced SREBP-1c activation and reduces hepatic steatosis in mice. J. Clin. Investig..

[38] Chen Y., Wu Z., Zhao S., Xiang R. (2016). Chemical chaperones reduce ER stress and adipose tissue inflammation in high fat diet-induced mouse model of obesity. Sci. Rep..

[39] Tocher D.R. (2003). Metabolism and functions of lipids and fatty acids in teleost fish. Rev. Fish. Sci..

[40] Li L., Xie P., Chen J. (2005). In vivo studies on toxin accumulation in liver and ultrastructural changes of hepatocytes of the phytoplanktivorous bighead carp ip-injected with extracted microcystins. Toxicon.

[41] Li L., Xie P., Chen J. (2007). Biochemical and ultrastructural changes of the liver and kidney of the phytoplanktivorous silver carp feeding naturally on toxic Microcystis blooms in Taihu Lake, China. Toxicon.

[42] Sozio M.S., Liangpunsakul S., Crabb D. (2010). The role of lipid metabolism in the pathogenesis of alcoholic and nonalcoholic hepatic steatosis. Semin. Liver Dis..

[43] Li X.Y., Chung I.K., Kim J.I., Lee J.A. (2004). Subchronic oral toxicity of microcystin in common carp (Cyprinus carpio L.) exposed to Microcystis under laboratory conditions. Toxicon.

[44] Li L., Xie P., Li S., Qiu T., Guo L. (2007). Sequential ultrastructural and biochemical changes induced in vivo by the hepatotoxic microcystins in liver of the phytoplanktivorous silver carp Hypophthalmichthys molitrix. Comp. Biochem. Physiol. Part C: Toxicol. Pharmacol..

[45] Bertolotti A., Zhang Y., Hendershot L.M., Harding H.P., Ron D. (2000). Dynamic interaction of BiP and ER stress transducers in the unfolded-protein response. Nat. Cell Biol..

[46] Shen J., Chen X., Hendershot L., Prywes R. (2002). ER stress regulation of ATF6 localization by dissociation of BiP/GRP78 binding and unmasking of Golgi localization signals. Dev. Cell.

[47] Malhotra J.D., Kaufman R.J. (2007). Endoplasmic reticulum stress and oxidative stress: A vicious cycle or a double-edged sword?. Antioxid. Redox Signal..

[48] Postic C., Girard J. (2008). The role of the lipogenic pathway in the development of hepatic steatosis. Diabetes Metab..

[49] Colgan S.M., Al-Hashimi A.A., Austin R.C. (2011). Endoplasmic reticulum stress and lipid dysregulation. Expert Rev. Mol. Med..

[50] Howarth D.L., Lindtner C., Vacaru A.M., Sachidanandam R., Tsedensodnom O., Vasilkova T., Buettner C., Sadler K.C. (2014). Activating transcription factor 6 is necessary and sufficient for alcoholic fatty liver disease in zebrafish. PLoS Genet..

[51] Gregor M.F., Hotamisligil G.S. (2007). Thematic review series: Adipocyte Biology. Adipocyte stress: The endoplasmic reticulum and metabolic disease. J. Lipid Res..

[52] Passeri M.J., Cinaroglu A., Gao C., Sadler K.C. (2009). Hepatic steatosis in response to acute alcohol exposure in zebrafish requires sterol regulatory element binding protein activation. Hepatology.

[53] Amemiya-Kudo M., Shimano H., Hasty A.H., Yahagi N., Yoshikawa T., Matsuzaka T., Okazaki H., Tamura Y., Iizuka Y., Ohashi K. (2002). Transcriptional activities of nuclear SREBP-1a,-1c, and-2 to different target promoters of lipogenic and cholesterogenic genes. J. Lipid Res..

[54] Huang C., Chen Q.L., Luo Z., Shi X., Pan Y.X., Song Y.F., Zhuo M.Q., Wu K. (2014). Time-dependent effects of waterborne copper exposure influencing hepatic lipid deposition and metabolism in javelin goby Synechogobius hasta and their mechanism. Aquat. Toxicol..

[55] Zhang J., Sun P., Kong T., Yang F., Guan W. (2016). Tributyltin promoted hepatic steatosis in zebrafish (Danio rerio) and the molecular pathogenesis involved. Aquat. Toxicol..

[56] Song Y.F., Luo Z., Zhang L.H., Hogstrand C., Pan Y.X. (2016). Endoplasmic reticulum stress and disturbed calcium homeostasis are involved in copper-induced alteration in hepatic lipid metabolism in yellow catfish Pelteobagrus fulvidraco. Chemosphere.

[57] Cao X.F., Dai Y.J., Liu M.Y., Yuan X.Y., Wang C.C., Huang Y.Y., Liu W.B., Jiang G.Z. (2019). High-fat diet induces aberrant hepatic lipid secretion in blunt snout bream by activating endoplasmic reticulum stress-associated IRE1/XBP1 pathway. Biochim. Biophys. Acta (BBA)-Mol. Cell Biol. Lipids.

[58] Horton J.D., Goldstein J.L., Brown M.S. (2002). SREBPs: Activators of the complete program of cholesterol and fatty acid synthesis in the liver. J. Clin. Investig..

[59] Lhoták Š., Sood S., Brimble E., Carlisle R.E., Colgan S.M., Mazzetti A., Dickhout J.G., Ingram A.J., Austin R.C. (2012). ER stress contributes to renal proximal tubule injury by increasing SREBP-2-mediated lipid accumulation and apoptotic cell death. Am. J. Physiol. Ren. Physiol..

[60] Rong S., Cortes V.A., Rashid S., Anderson N.N., McDonald J.G., Liang G., Moona Y.A., Hammerb R.E., Horton J.D. (2017). Expression of SREBP-1c requires SREBP-2-mediated generation of a sterol ligand for LXR in livers of mice. Elife.

[61] Kuo Y.T., Lin T.H., Chen W.L., Lee H.M. (2012). Alpha-lipoic acid induces adipose triglyceride lipase expression and decreases intracellular lipid accumulation in HepG2 cells. Eur. J. Pharmacol..

[62] Lin W., Hou J., Guo H., Li L., Wang L., Zhang D., Li D., Tang R. (2018). The synergistic effects of waterborne microcystin-LR and nitrite on hepatic pathological damage, lipid peroxidation and antioxidant responses of male zebrafish. Environ. Pollut..

[63] Hou J., Su Y., Lin W., Guo H., Li L., Anderson D.M., Li D., Tang R., Chi W., Zhang X. (2018). Estrogenic potency of MC-LR is induced via stimulating steroidogenesis: In vitro and in vivo evidence. Environ. Pollut..

[64] Bradford M.M. (1976). A rapid and sensitive method for the quantitation of microgram quantities of protein utilizing the principle of protein-dye binding. Anal. Biochem..

[65] Livak K.J., Schmittgen T.D. (2001). Analysis of relative gene expression data using real-time quantitative PCR and the 2^−ΔΔCT^ method. Methods.

