# Supplementary material for: Chronic Microcystin-LR Exposure Induces Abnormal Lipid Metabolism via Endoplasmic Reticulum Stress in Male Zebrafish
Source: Toxins (Basel). 2020 Feb 7;12(2):107. doi: 10.3390/toxins12020107 (PMC7076763; doi:10.3390/toxins12020107)
Supplement: Supplementary file 1 [file toxins-12-00107-s001.pdf]

Supplementary Materials: Chronic Microcystin-LR exposure induces abnormal lipid metabolism via endoplasmic reticulum stress in male zebrafish

Dandan Zhang, Wang Lin, Yinjie Liu, Honghui Guo, Lingkai Wang, Liping Yang, Li Li, Dapeng Li and Rong Tang

Table S1. Transcriptional levels of genes after exposed to MC-LR[a.]

Gene	0 μg/L	1 μg/L	5 μg/L	25 μg/L
atf6	1.06 ± 0.19	2.14 ± 0.11**	2.20 ± 0.12**	3.11 ± 0.04**
atf4b1	1.00 ± 0.05	1.00 ± 0.10	0.97 ± 0.10	1.11 ± 0.09
ern1	1.00 ± 0.03	2.61 ± 0.18**	2.78 ± 0.10**	1.46 ± 0.14*
xbp1s	1.02 ± 0.12	1.26 ± 0.29	2.90 ± 0.28**	2.31 ± 0.36**
eif2s1	1.01 ± 0.09	1.01 ± 0.10	1.18 ± 0.14	1.03 ± 0.06
eif2ak3	1.00 ± 0.03	1.70 ± 0.14**	1.70 ± 0.02**	1.66 ± 0.26**
mapk8	1.01 ± 0.07	1.64 ± 0.24	2.65 ± 0.37**	1.71 ± 0.46
chop	1.01 ± 0.06	1.41 ± 0.06*	1.00 ± 0.16	0.70 ± 0.06
hspa5	1.05 ± 0.16	1.25 ± 0.27	2.01 ± 0.11**	2.40 ± 0.20**
srebf1	1.00 ± 0.04	0.99 ± 0.12	2.44 ± 0.09**	1.66 ± 0.02**
acaca	1.05 ± 0.17	1.27 ± 0.20	2.38 ± 0.11**	2.30 ± 0.52*
fasn	1.08 ± 0.25	1.32 ± 0.16	1.37 ± 0.07	1.47 ± 0.10
scd	1.01 ± 0.11	1.40 ± 0.10	2.73 ± 0.10**	2.48 ± 0.22**
srebf2	1.00 ± 0.02	1.70 ± 0.17**	1.80 ± 0.21**	1.59 ± 0.03*
hmgcs1	1.01 ± 0.08	1.42 ± 0.13*	1.83 ± 0.14**	1.76 ± 0.07**
hmgcra	1.01 ± 0.07	1.63 ± 0.05	2.75 ± 0.46**	2.71 ± 0.40**
atgl	1.00 ± 0.03	0.41 ± 0.08**	0.68 ± 0.06**	0.46 ± 0.05**
hsla	1.01 ± 0.08	0.45 ± 0.06**	0.69 ± 0.01*	0.39 ± 0.12**
cpt1aa	1.10 ± 0.27	0.50 ± 0.17*	0.53 ± 0.07	0.42 ± 0.18*

[a] Value represent means ± SE of 5 replicates of each treatment and are expressed as fold change. Asterisk * and ** indicate significant differences at $p< 0.05$ and $p < 0.01$ between MC-LR treated groups and the control group, respectively.

Table 2. Sequences of primers for tested genes a.

Target gene	Primer sequences (from 5′to 3′)	Accession number	Product length (bp)	Amplification efficiency (%)
gapdh	F : CTGGTGACCCGTGCTGCTT<br>R : TTTGCCGCCTTCTGCCTTA	NM_001115114	150	98
atf6	F : CTGTGGTGAAACCTCCACCT<br>R : CATGGTGACCACAGGAGATG	NM_001110519	200	96
atf4b1	F : TGGCGTCTCTCCTAGCAAAC<br>R : GAGAAGCTGCGGTATTTGCG	XM_005172055	80	102
ern1	F : ATGGGTAAGAAGCAGGATGTG<br>R :CAGGGACGAAGATGGACATAAC	XM_001020530	108	89
xbp1s	F : ACCAGTTAAGCTGGAAGCCC<br>R : GTTTCAGAGAAGGCGACGGA	NM_131874	132	110
eif2s1	F : CCAAAGATGAGCAGCTGGAGA<br>R : ATCCGACACAGCCTGCTTAAA	NM_199569	113	93
eif2ak3	F : TGGGCTCTGAAGAGTTCGAT<br>R : TGTGAGCCTTCTCCGTCTTT	XM_005156585	193	95
mapk8	F : TGCTACGTCGTCTTGCCATT<br>R : CGTGCCATTTAATGAACCTCCA	NM_131721	142	91

chop	F : GAGGACACGTAGAGAAGGGG<br>R : TCCGTTGAGCTCCACATTCTT	NM_001082825	196	95
hspa5	F : TTCTTCTGGACGTGTGTCCG<br>R : TTGTCTTTGGTCAGGGGACG	NM_213058	184	97
srebf1	F : CATCCACATGGCTCTGAGTG<br>R : CTCATCCACAAAGAAGCGGT	NM_001105129	250	110
acaca	F : GGACGGACCCTTGCACAATA<br>R : CCTCTGCAGGTCGATACGTC	NM_001271308	91	108
fasn	F : GAGAAAGCTTGCCAAACAGG<br>R : GAGGGTCTTGCAGGAGACAG	XM_009306806	203	91
scd	F : TTCTGGCCATCGGAAACTCC<br>R : TCTCTCGATGACTTCCGGGT	NM_198815	179	108
srebf2	F : CACTCACACAAGCACACACG<br>R : ACCTGGTTCTGGATGAATCG	NM_001089466	208	102
hmgcs1	F : AACTGCTGCTCAGAGTCGTC<br>R : GGGATGCTTCGAGGTTCGAT	NM_201085	165	111
hmgcra	F : CTGAGGCTCTGGTGGACGTG<br>R : GCAGCTACGATGTTGGCG	NM_001079977	103	98
atgl	F : ACACACTTACACCGCGTGAT<br>R : AGCACGTTTTCTCCATCCGT	XM_005174256	98	111
hsla	F : AGGTAAGCAAAGGTTGTCCGA<br>R : TTCATGACCCCCAACAGACG	NM_001316725	147	103
cpt1aa	F : TCTACCTGAGAGGTCGTGGG<br>R : TGACGTTTCCTGCTCTTGCT	NM_001044854	108	90

[a] *gapdh*, glyceraldehyde-3-phosphate dehydrogenase ; *atf6*, activating transcription factor 6 ; *atf4b1*, activating transcription factor 4a; *ern1*, endoplasmic reticulum to nucleus signaling 1 ; *xbp1s*, X-box binding prot ein 1; *eif2s1*, eukaryotic translation initiation factor 2; *eif2ak3*, eukaryotic translation initiation factor 2-alpha kinase 3; *mapk8*, mitogen-activated protein kinase 8 ; *chop*, DNA damage inducible transcript 3; *hspa5*, heat shock protein 5; *srebf1*, sterol regulatory element binding transcription factor 1; *acaca*, acetyl-CoA carboxylase alpha; *fasn*, fatty acid synthase; *scd*, stearoyl-CoA desaturase; *srebf2*, sterol regulatory element binding transcription factor 2; *hmgcs1*, 3-hydroxy-3-methylglutaryl-CoA synthase 1; *hmgcra*, 3-hydroxy-3-methylglutaryl-CoA reductase a; *atgl*, patatin-like phospholipase domain containing 2; *hsla*, hormone-sensitive a; *cpt1aa*, carnitine palmitoyltransferase 1Aa.
